# *EGFR*突变NSCLC患者TKIs耐药后ICIs治疗进展

**DOI:** 10.3779/j.issn.1009-3419.2022.101.37

**Published:** 2022-08-20

**Authors:** 然 陈, 昌 江, 丽 马, 树才 张

**Affiliations:** 1 441000 襄阳，湖北医药学院附属襄阳市第一人民医院肿瘤科 Department of Oncology, Xiangyang No.1 People's Hospital, Hubei University of Medicine, Xiangyang 441000, China; 2 101149 北京，首都医科大学附属北京胸科医院，北京市结核病胸部肿瘤研究所肿瘤内科 Department of Medical Oncology, Beijing Chest Hospital, Capital Medical University, Beijing Tuberculosis and Thoracic Tumor Research Institute, Beijing 101149, China

**Keywords:** *EGFR*突变, EGFR-TKIs耐药, 肺肿瘤, 免疫检查点抑制剂, 免疫治疗, *EGFR* mutations, EGFR-TKIs resistance, Lung neoplasms, Immune checkpoint inhibitors, Immunotherapy

## Abstract

表皮生长因子受体酪氨酸激酶抑制剂（epidermal growth factor receptor-tyrosine kinase inhibitors, EGFR-TKIs）耐药后的*EGFR*突变晚期非小细胞肺癌（non-small cell lung cancer, NSCLC）患者的后续治疗已成为目前的热点和难点。免疫检查点抑制剂（immune checkpoint inhibitors, ICIs）治疗对于这部分患者来说是一个崭新而重要的选择，然而众多研究却显示疗效差强人意。但同时，国内外也有研究表明部分驱动基因阳性且靶向治疗耐药后的患者接受ICIs联合治疗仍显效。那么，在免疫治疗时代，不同的患者接受不同的ICIs联合策略的疗效有何差异？影响疗效的因素有哪些？这些因素与其他免疫治疗疗效预测标志物的内在联系有哪些？这些问题都有着广阔而重要的研究价值。

## Abstract (en)

The follow-up treatment of patients with advanced non-small cell lung cancer (NSCLC) with epidermal growth factor receptor (*EGFR*) mutation after drug resistance to EGFR-tyrosine kinase inhibitors (TKIs) have become a hotspot and difficulty at present. Immune checkpoint inhibitors (ICIs) therapy is a new and important choice for these patients, but many studies have shown unsatisfactory efficacy. However, some domestic and foreign studies have shown that ICIs combination therapy is still effective in some patients with positive driver genes and drug resistance after targeted therapy. So, in the era of immunotherapy, what are the differences in the efficacy of different combination immunotherapy strategies for different patients? What are the factors that affect efficacy? What are the interrelationships between these factors and other immunotherapy efficacy prediction biomarkers? All these problems have broad and important research value.

## 1 背景、现状及目的

非小细胞肺癌（non-small cell lung cancer, NSCLC）分子分型的出现为晚期NSCLC的治疗开创了全新的局面。其中，以驱动基因表皮生长因子受体（epidermal growth factor receptor, *EGFR*）突变为代表，陆续研发的EGFR-酪氨酸激酶抑制剂（*EGFR*-tyrosine kinase inhibitors, EGFR-TKIs）显著延长了这部分患者的无进展生存期（progression-free survival, PFS）及总生存期（overall survival, OS）^[1]^。但是，接受EGFR-TKIs治疗的这部分晚期NSCLC患者多数会在治疗后的8个月-13个月发生耐药^[2]^，耐药后的治疗方法有限且疗效不确切。一代、二代EGFR-TKIs耐药群体中约50%的患者会出现继发*EGFR* T790M突变^[3]^，后续可接受三代EGFR-TKIs治疗。然而，对于一代、二代EGFR-TKIs治疗进展后未出现*EGFR* T790M突变以及三代EGFR-TKIs耐药后的患者而言，目前尚无明确的标准治疗方案，其后续的治疗策略已成为目前的热点和难点问题。

随着免疫治疗时代的来临，以ICIs治疗为代表的免疫治疗在驱动基因阴性的NSCLC患者中取得重大的突破，其治疗模式从单药扩展至联合，为肺癌患者提供了更多的选择。多种ICIs治疗策略已被证实可以使晚期NSCLC患者得到生存获益^[4-7]^，但其获益人群有着较大的局限性。在未经选择的人群中只有20%患者能从中获益^[7]^。其中*EGFR*突变型晚期NSCLC患者在目前的ICIs治疗中的疗效及其相关影响因素的临床研究结果更是不够明朗。

自2018年以来，不乏有研究者们寄希望于将ICIs治疗应用到*EGFR*突变型NSCLC人群中^[8, 9]^，但目前鲜有见到令人振奋的阳性结果。前期研究^[10]^表明：由于*EGFR*突变的人群生物学特性不同，驱动型癌基因导致免疫抑制激活的通路较多，免疫微环境存在较多抑制信号，*EGFR*突变人群一度被认为是“免疫豁免”人群。一些临床试验和*meta*分析^[9, 11]^也提示ICIs治疗*EGFR*突变人群的获益有限，疗效劣于EGFR野生型患者，其获益程度与化疗类似，甚至略低于化疗，相关权威指南及大型临床试验也纷纷将这部分*EGFR*突变型NSCLC患者排除在ICIs治疗的人群之外，已成为目前亟待解决的问题。

本文从临床视角出发，关注*EGFR*突变型晚期NSCLC患者接受ICIs治疗的这一人群，从以下方面进行综述：①探寻影响*EGFR*突变的晚期NSCLC患者ICIs治疗疗效的相关因素；②探讨与ICIs的最佳联合应用策略；③筛选能从ICIs治疗中获益的优势群体。这些问题都是当下ICIs治疗应用和发展中的重要环节。我们从前瞻性临床试验结果到潜在的治疗策略进行综述，以期为*EGFR*突变NSCLC患者的ICIs治疗策略和全程管理提供一定的理论基础和客观依据。

## 2 *EGFR*突变型NSCLC与ICIs治疗疗效相关的主要影响因素

### 2.1 *EGFR*突变与程序性死亡蛋白配体1（programmed cell death ligand 1, PD-L1）表达的关系

PD-L1作为程序性死亡蛋白受体1（programmed cell death 1, PD-1）的配体，与T细胞表面的PD-1结合可以抑制T细胞的激活，使肿瘤细胞发生免疫逃逸，促进肿瘤发展^[12]^，是目前NSCLC患者ICIs治疗中最常用的生物标记物和最重要的疗效预测指标。然而，随着研究的深入，发现肿瘤驱动基因通路和PD-1/PD-L1通路可能存在相互作用，肺癌驱动基因突变与PD-1/PD-L1信号的异常激活之间有着密切的联系。*EGFR*突变肺癌PD-L1表达水平增高还是降低在不同的临床前研究和临床研究结果中并不一致。

#### 2.1.1 临床前研究

细胞和动物实验表明*EGFR*突变的肺癌细胞PD-L1表达显著高于野生型肺癌细胞。Akbay等^[13]^在2013年通过小鼠模型发现*EGFR*突变可诱导PD-L1表达，促进免疫抑制性微环境的形成，而PD-1单抗能明显抑制*EGFR*突变肿瘤的生长并延长生存期。该研究首次揭示了肺癌*EGFR*突变可激活PD-1/PD-L1通路以促进肿瘤免疫逃逸。随后，很多研究从不同角度证实了*EGFR*突变可上调PD-1/PD-L1的表达并阐释了相关调节机制，其中有众多体内外试验^[14-18]^证明了*EGFR*突变通过不同的信号通路调节PD-L1的表达。

#### 2.1.2 临床研究

在临床标本的研究中，*EGFR*突变与PD-L1表达水平之间的关联存在一些争议。Azuma等^[19]^通过检测164例NSCLC手术切除标本PD-L1表达水平，首次报道*EGFR*突变具有较高的PD-L1免疫组化评分，且PD-L1高表达预示着预后不良。然而，也有研究得出与此不同的结论，Takada等^[20]^通过对441例手术切除的肺腺癌标本进行免疫组化染色，发现PD-L1表达中有21%与野生型EGFR显著相关。一项汇总分析^[21]^纳入18个研究的3, 969例患者，发现*EGFR*突变型NSCLC的PD-L1表达较野生型更低，比值比（odds ratio, OR）为0.59（95%CI: 0.39-0.92, *P* < 0.02）。

临床研究的结论之所以存在争议的原因可能包括以下方面：各临床研究中不同PD-L1检测抗体的应用、临床研究中不同人群的选择、免疫组织化学（immunohistochemistry, IHC）技术及评分标准存在差异、肿瘤细胞PD-1表达与免疫细胞PD-L1表达之间的差异等。此外，一些临床因素如肿瘤异质性、检测前的既往治疗对PD-L1表达的影响以及患者基线特征等都会影响最终的结论。

### 2.2 PD-L1与EGFR-TKIs疗效的关系

EGFR-TKIs的疗效与其原发性耐药和获得性耐药直接相关。PD-L1作为一种免疫抑制分子，不仅与*EGFR*基因突变之间存在调节关系，而且PD-L1的表达和EGFR-TKIs耐药也显示出新的相关性^[20, 22]^。免疫逃逸和靶向耐药之间的联系已经在黑色素瘤模型中得到了证实^[23]^，在肺腺癌中也已观察到EGFR-TKIs持续处理的耐药细胞中PD-L1表达上调^[22]^。

在EGFR-TKIs获得性耐药中，有研究^[24]^显示，在EGFR-TKIs治疗前，携带*EGFR*突变的肺癌细胞不具有PD-L1蛋白高表达，而在诱导细胞产生获得性耐药之后，PD-L1的表达水平显著升高。

在EGFR-TKIs原发性耐药中，虽然目前已经明确EGFR-TKIs获得性耐药的部分机制，但对原发性耐药的机制却知之甚少。原发性耐药机制被认为是高度异质性的，此外，*BIM*（Bcl-2 interacting mediator of cell death）基因缺失多态性、*EGFR*基因外显子20的插入等都可能参与了原发性耐药的形成^[25]^。有研究^[26]^表明，PD-L1强阳性表达与EGFR-TKIs的原发性耐药和不良反应有关。HSU等^[27]^进一步探讨了PD-L1表达水平与原发性耐药之间的关系，发现患者PD-L1表达水平越高，原发性耐药发生率也越高。Zhang等^[28]^发现，PD-L1在EGFR-TKIs原发性耐药中起重要作用，认为PD-L1可能通过激活转化生长因子β（transforming growth factor beta, TGF-β）/受体激活型Smad（receptor-activated Smad, R-Smad）信号通路诱导细胞发生上皮间质转化（epithelial-mesenchymal transition, EMT），进而导致产生对吉非替尼的原发性耐药。

临床诊疗中，对于晚期初诊非鳞NSCLC患者来说，驱动基因的检测是重中之重，不论PD-L1表达状态如何，EGFR-TKIs是晚期*EGFR*突变患者的标准一线治疗。有研究^[21]^显示，尽管*EGFR*突变患者的PD-L1表达率相较总体人群偏低，但仍有一定比例患者存在驱动基因突变且PD-L1高表达。在一项针对101例*EGFR*敏感突变阳性的NSCLC患者研究^[26]^中，显示PD-L1的高表达显著降低了EGFR-TKIs药物治疗的客观缓解率（objective response rate, ORR）（35.7%），缩短了PFS（3.8个月）。

### 2.3 *EGFR*突变NSCLC患者接受ICIs治疗疗效与肿瘤突变负荷（tumor mutation burden, TMB）的关系

TMB是一种新型基因标志物，可反映肿瘤基因突变的总体状况^[29]^，已广泛应用于ICIs治疗的获益人群筛选^[30]^及疗效预测^[31]^，在*EGFR*突变型NSCLC中也有相应的探索性研究。

#### 2.3.1 高TMB水平预示着良好的ICIs治疗疗效

作为优势驱动基因，*EGFR*突变型患者在免疫治疗中总体获益不大，这与*EGFR*突变型患者肿瘤微环境肿瘤微环境（tumor microenvironment, TME）的弱免疫原性、TMB低水平的特征有关^[32]^。临床前研究^[11]^显示：约20, 000个黑色素瘤样本中发现的20种新抗原，其中只有8%的新抗原来自驱动基因突变，另外92%的新抗原全部来自非驱动基因突变。而新抗原的产生与TMB密切相关，是激活特异性T淋巴细胞介导的抗肿瘤效应的重要介质，这也是驱动基因突变肿瘤患者免疫治疗效果不佳的重要原因之一。也有一些研究探讨了*EGFR*突变型患者后线免疫治疗的可能性，并提出TMB高表达是生存获益的潜在机制。一项日本的研究^[33]^探索了EGFR-TKIs耐药后Nivolumab的疗效，亚组分析显示，9例已经评估过TMB值的患者中，有应答的3例TMB值显著高于无应答的6例（*P*=0.038），由此推测高TMB很可能是Nivolumab获益的原因。

#### 2.3.2 不同*EGFR*突变亚型存在不同的TMB水平导致明显差异的ICIs治疗反应

Hastings等^[34]^评估了126例*EGFR*经典突变患者的ICIs疗效与预后，发现19del突变患者ORR（7% *vs* 22%, *P*=0.002）和OS（HR=0.69, 95%CI: 0.493-0.965, *P*=0.03）都显著差于L858R突变，这与19del突变患者TMB水平更低有关。另外，该研究还发现，L858R突变与EGFR野生型的ORR（16% *vs* 22%, *P*=0.42）和OS（HR=0.917, 95%CI: 0.597-1.409, *P*=0.69）相似。Haratani等^[33]^纳入了25例二线接受Nivolumab治疗的EGFR-TKIs耐药患者，T790M阴性耐药患者的中位PFS长于T790M突变患者（HR=0.48, 95%CI: 0.20-1.24, *P*=0.099）。综上提示：*EGFR* L858R突变及*EGFR* T790M野生型的NSCLC患者更有可能从免疫治疗中获益。首都医科大学北京胸科医院马丽教授团队的一项研究^[35]^收集了27例经靶向治疗进展后接受了ICIs联合化疗以及抗血管生成药物治疗的患者，研究结果发现：*EGFR*突变患者人群能从后线ICIs联合治疗中获益，但合并T790M突变的患者后线接受ICIs联合治疗疗效差，研究结果也与Haratani的观点吻合。

尽管美国国立综合癌症网络（National Comprehensive Cancer Network, NCCN）指南不推荐*EGFR*突变型患者行ICIs治疗，但以上研究均证实，EGFR-TKIs耐药后的NSCLC患者仍可从ICIs治疗中获益，尤其是T790M阴性获得性突变及L858R突变的患者。这两类突变亚型均是*EGFR*突变中TMB相对较高的，因此高TMB可指导ICIs在*EGFR*突变型NSCLC中的应用。

### 2.4 外周血细胞因子

细胞因子是一类大多由免疫细胞合成、分泌的可在细胞间传递信息、具有免疫调节和效应功能的低分子质量蛋白或多肽，通过调节免疫反应的强度和持续时间在免疫系统中起重要作用。在一项纳入63例接受PD-1抗体治疗的晚期恶性黑色素瘤和晚期肺癌患者的研究^[36]^中，通过对比PD-1抗体使用前和使用2周-4周后血液中白细胞介素（interleukin, IL）-8的变化，可以较早地预测PD-1抗体的疗效，甚至可以辅助判断“假进展”，该研究界定浓度变化阈值为9.2%。另一项研究^[37]^发现，血浆IL-6和吲哚胺2, 3-双加氧酶（indoleamine 2, 3-dioxygenase, IDO）在有明显炎症状态的TME中升高，提示其可能具有潜在的疗效预测价值。

### 2.5 其他与*EGFR*突变NSCLC患者ICIs治疗相关的标志物

TME是肿瘤细胞赖以生存的内环境，TME在增强或抑制肿瘤免疫应答中发挥了重要的作用。*EGFR*突变可以调节肿瘤浸润淋巴细胞（tumor-infiltrating lymphocytes, TILs）^[26, 38]^、调节性T细胞（regulatory T cells, Tregs）^[34, 39, 40]^、肿瘤相关巨噬细胞（tumor-associated macrophages, TAMs）^[41]^和髓系来源抑制性细胞（myeloid derived suppressor cell, MDSC）^[41]^等免疫细胞及趋化因子^[40, 42]^以重塑TME，在免疫耐受和免疫逃逸过程中发挥重要作用。

#### 2.5.1 TILs

TILs是指离开血循环进入到肿瘤中的淋巴细胞，是肿瘤微环境中最简单的免疫活性测量计数。研究^[43]^报道TILs与多种肿瘤预后有关，尤其是肺癌，已有研究建议将TILs评分加入到新的肿瘤原发灶-淋巴结-转移（tumor-node-metastasis, TNM）分期中。越来越多的研究^[44]^表明：TILs的存在有助于使罹患NSCLC、EBV^+^胃癌以及黑色素瘤的人群在免疫治疗中获益，此外有临床研究^[45]^表明，尤其是以CD8^+^ T细胞为主的TILs，更有助于患者在免疫治疗中获益。

#### 2.5.2 Tregs

Tregs是一类控制体内自身免疫反应性的T细胞亚群，Treg浸润增多则免疫抑制功能增强。Sugiyama等^[46]^用流式细胞术检测26例手术切除的肺腺癌标本，发现*EGFR*突变腺癌中肿瘤浸润Treg细胞的数量及Treg细胞/CD8^+^ T细胞比值明显高于EGFR野生型腺癌。

## 3 ICIs相关治疗策略在*EGFR*突变型NSCLC中的探索

### 3.1 ICIs单药治疗

CheckMate 057研究^[7]^是一项随机双盲的Ⅲ期临床研究，主要分析了二线使用纳武单抗或多西他赛单药治疗晚期非鳞NSCLC患者的效果差异。该研究纳入了82例既往接受过EGFR-TKIs或含铂两药方案治疗进展的*EGFR*突变晚期非鳞NSCLC患者。亚组分析结果显示：纳武单抗二线治疗对比多西他赛化疗并无生存获益，而EGFR野生型亚组中患者接受ICIs治疗后中位生存期则明显延长。KEYNOTE-010研究^[47]^再次证实了上述观点，其亚组分析结果显示85例*EGFR*突变晚期NSCLC患者无法从ICIs单药治疗中获得OS优势（HR=0.88, 95%CI: 0.45-1.70）。以上均提示：在*EGFR*突变型NSCLC二线ICIs单药治疗中K药和O药均无获益。

另外一项有关PD-1抑制剂O药单药治疗的Ⅱ期临床WJOG8515L研究^[48]^在*EGFR*阳性（非继发T790M突变）、EGFR-TKIs耐药后的102例非鳞NSCLC患者中对比了Nivolumab单药组与卡铂/培美曲塞化疗组的疗效和安全性，结果显示：O药组（*n*=52）的中位PFS和OS分别为1.7个月和20.7个月，而化疗组（*n*=50）的中位PFS和中位OS分别为5.6个月和19.9个月。O药组的ORR和缓解持续时间（duration of response, DOR）分别为9.6%和5.3个月，而化疗组的ORR和DOR分别为36.0%和5.5个月。研究结论：在后线治疗中，O药不优于化疗。

ATLANTIC研究^[49]^是一项基于亚、欧、北美139个研究中心，针对晚期NSCLC三线之后ICIs（Durvalumab单药）治疗的开放性Ⅱ期大型单臂临床研究结果显示：其队列1（*EGFR*/*ALK*阳性患者）中*EGFR*阳性患者OS为16.1个月，数值上优于*ALK*阳性组的6.3个月。进行PD-L1表达分层后显示：*EGFR*/*ALK*阳性PD-L1表达 > 25%的患者人群中接受Durvalumab单药治疗仍有12.2%的三线有效率。

由此可见，在EGFR阳性、TKIs耐药后NSCLC的ICIs单药后线治疗中，PD-1抑制剂（包括K药和O药）的疗效相对于化疗并未见到有获益趋势，然而在PD-L1抑制剂（I药）中的尝试，三线的ORR结果似乎让我们看到了一丝曙光。

### 3.2 ICIs联合治疗

#### 3.2.1 ICIs联合EGFR-TKIs

CheckMate 012研究^[50]^评估了Nivolumab联合厄洛替尼二线治疗*EGFR*突变晚期NSCLC的安全性与有效性。20例EGFR-TKIs经治患者的ORR为15%，中位PFS和中位OS分别为5.1个月和18.7个月。所有入组患者都出现了治疗相关不良反应（treatment-related adverse event, trAE），5例患者出现3级trAE，其中2例因不良反应停药。TATTON研究^[51]^是一项IB期临床研究，主要探索奥希替尼联合Durvalumab治疗*EGFR*突变晚期NSCLC患者的最佳剂量和安全性。该研究中期发现，联合治疗组患者间质性肺炎的发生率明显升高，甚至高达38%（13/34）。因此，该研究的后期Ⅲ期随机对照研究CAURAL（NCT02454933）^[52]^提前关组。该研究最终只纳入了29例*EGFR*突变晚期NSCLC患者，研究结果显示：接受奥希替尼联合Durvalumab治疗的*EGFR* T790M突变晚期NSCLC患者的ORR低于接受奥希替尼单药治疗的患者（64% *vs* 80%）。

由此可见，ICIs联合EGFR-TKIs未明显提高疗效，反而显著增加了毒副作用，不同药物组合之间的毒性谱也存在差异，表明目前尚无循证医学证据联合EGFR-TKIs和不同的PD-1/PD-L1抑制剂。此外，联合治疗中不良反应的发生机制有待进一步研究。

#### 3.2.2 ICIs联合化疗/抗血管生成治疗

多项基础研究^[53-57]^表明，化疗可以降低调节性T细胞的活性，增强肿瘤抗原的交叉呈递，并降低肿瘤细胞中PD-L1的表达水平。另外，大量的临床前模型^[41, 58]^验证，抗血管抑制剂联合ICIs治疗NSCLC具有协同增效的作用。因此理论上，化疗/抗血管治疗联合ICIs治疗具有协同抗肿瘤的作用。

近年来，随着国内生物制药水平的提高，涌现出一批优秀的PD-1抑制剂产品。其中有关信迪利单抗的ORIENT31研究^[59]^，是首次在全球范围内证实PD-1抑制剂联合抗血管药物以及化疗在EGFR-TKIs治疗进展的*EGFR*突变非鳞NSCLC人群中显著提高PFS的前瞻性、双盲、多中心Ⅲ期临床研究。研究结果显示：在意向治疗（intention-to-treat analysis, ITT）人群中，信迪利单抗联合贝伐珠单抗及化疗（A组），对比化疗（C组）获得了显著且具有临床意义的PFS延长（中位PFS：6.9个月 *vs* 4.3个月，HR=0.464，95%CI: 0.337-0.639，*P* < 0.000, 1）。

国内另一种PD-1抑制剂特瑞普利单抗的一项前瞻性、多中心、开放性、单臂Ⅱ期研究（NCT03513666）^[60]^显示，特瑞普利单抗联合化疗在EGFR-TKIs治疗失败的*EGFR*突变阳性且T790M阴性的晚期NSCLC患者中的疗效结果显示：联合组ORR达50%，疾病控制率（disease control rate, DCR）达87.5%，中位DOR为7.0个月，整体人群PFS达7.0个月，PD-L1表达阳性患者PFS可达8.3个月，且3级以上免疫相关不良事件发生率仅7.5%。

IMpower150研究^[61]^是首个也是目前唯一一个证实免疫治疗联合抗血管生成治疗和化疗模式一线治疗转移性非鳞NSCLC，PFS及OS均得到显著获益阳性结果的临床Ⅲ期研究。研究发现在ICIs联合组中，回顾EGFR/ALK^+^这部分人群，最终可以得到一定程度的获益。研究结果表明接受阿特珠单抗联合贝伐珠单抗及化疗治疗的*EGFR*/*ALK*突变阳性晚期非鳞NSCLC患者的中位PFS长于接受化疗联合贝伐珠单抗治疗的患者中位PFS（9.7个月 *vs* 6.1个月，HR=0.59，95%CI：0.37-0.94，*P*=0.025, 3），OS较化疗联合抗血管生成治疗的患者显著延长（29.4个月 *vs* 18.1个月，HR=0.6，95%CI：0.31-1.41），这一国际多中心研究颠覆了人们对“*EGFR*突变免疫豁免”的理论，给*EGFR*突变NSCLC的ICIs治疗带来了新的希望。基于此类研究结果，其他ICIs联合化疗或联合抗血管生成药物的临床研究如KEYNOTE-789研究及CheckMate 722研究等已经陆续开展，都在探索EGFR-TKIs治疗进展后NSCLC患者接受ICIs联合治疗的获益方案。

## 4 总结与展望

免疫治疗的出现，确切地说，ICIs的出现极大程度地改变了NSCLC的治疗格局，但在*EGFR*突变NSCLC患者的研究和应用中却历经坎坷。

在*EGFR*突变NSCLC患者ICIs治疗疗效预测方面，目前除了既往研究较多的PD-L1表达、TMB展现出重要的预测价值外，肿瘤免疫微环境相关细胞因子也存在潜在的预测应用价值。另外，基因突变的亚型，如*EGFR* T790M阴性获得性突变及L858R突变的患者预示着较好的ICIs治疗预后。越来越多的研究表明单一的标志物可能无法准确预测疗效，寻找新的标志物、探索多种有效预测因子并构建预测模型将会是一个值得思考的方向。

在*EGFR*突变NSCLC患者ICIs治疗策略探索中，靶向治疗失败后ICIs单药（PD-1抑制剂）治疗对于初治和经治人群的获益都非常有限，但PD-L1抑制剂单药治疗似乎能带来获益，值得进一步探究；ICIs联合EGFR-TKIs不仅疗效不确定而且会出现不可耐受的毒性反应；ICIs联合化疗、抗血管生成治疗可能提高*EGFR*阳性患者获益，无论是国内有关PD-1抑制剂的联合方案还是国外的PD-L1抑制剂联合方案，均能给*EGFR*阳性晚期NSCLC患者带来获益，是当下值得推崇和探讨的思路。

另外，在*EGFR*敏感突变的晚期NSCLC靶向治疗中，PD-L1的高表达可能是EGFR-TKIs疗效预测的负面因素，此特点有助于我们在PD-L1高表达的NSCLC全程管理中及时调整治疗策略。
